# Neuroprotective effects of polyacrylic acid (PAA) conjugated cerium oxide against hydrogen peroxide- and 6-OHDA-induced SH-SY5Y cell damage

**DOI:** 10.1038/s41598-023-45318-6

**Published:** 2023-10-28

**Authors:** Rugmani Meenambal, Tomasz Kruk, Jacek Gurgul, Piotr Warszyński, Danuta Jantas

**Affiliations:** 1 Department of Experimental Neuroendocrinology, Maj Institute of Pharmacology Polish Academy of Sciences, Kraków, Poland; 2 Jerzy Haber Institute of Catalysis and Surface Chemistry, Polish Academy of Sciences, Kraków, Poland

**Keywords:** Cellular neuroscience, Parkinson's disease, Nanoparticles

## Abstract

Cerium oxide nanoparticles have been widely investigated against neurodegenerative diseases due to their antioxidant properties that aid in quenching reactive oxygen species. In this study, polyacrylic acid conjugated cerium oxide (PAA-CeO) nanoparticles were synthesized in a 50–60 nm size range with a zeta potential of − 35 mV. X-ray photoelectron spectroscopy analysis revealed a mixed valence state of Ce^4+^ and Ce^3+^. PAA-CeO nanoparticles were safe for undifferentiated (UN-) and retinoic acid-differentiated (RA-) human neuroblastoma SH-SY5Y cells and reduced the extent of cell damage evoked by hydrogen peroxide (H_2_O_2_) and 6-hydroxydopamine (6-OHDA). In the H_2_O_2_ model of cell damage PAA-CeO did not affect the caspase-3 activity (apoptosis marker) but attenuated the number of propidium iodide-positive cells (necrosis marker). In the 6-OHDA model, nanoparticles profoundly reduced necrotic changes and partially attenuated caspase-3 activity. However, we did not observe any impact of PAA-CeO on intracellular ROS formation induced by H_2_O_2_. Further, the flow cytometry analysis of fluorescein isothiocyanate-labeled PAA-CeO revealed a time- and concentration-dependent cellular uptake of nanoparticles. The results point to the neuroprotective potential of PAA-CeO nanoparticles against neuronal cell damage induced by H_2_O_2_ and 6-OHDA, which are in both models associated with the inhibition of necrotic processes and the model-dependent attenuation of activity of executor apoptotic protease, caspase-3 (6-OHDA model) but not with the direct inhibition of ROS (H_2_O_2_ model).

## Introduction

Increased oxidative stress being a result of reactive oxygen species (ROS) overproduction and/or inefficiency of the intracellular antioxidant defense system, is one of the main causes of the development and progression of several neurodegenerative diseases (ND), including the most common Alzheimer's disease (AD) and Parkinson's disease (PD)^1^. ROS are extremely detrimental to cells by disrupting their homeostasis and structural integrity. They could evoke lipid peroxidation, mitochondrial dysfunction, impairments in calcium signaling, and protein tyrosine nitration leading to neuronal cell death through apoptotic or non-apoptotic mechanisms^2,3^.

The excessive intracellular production of hydrogen peroxide (H_2_O_2_) is a well-known endogenous source of oxidative stress and is widely used to induce oxidative stress in different cell types, such as neuronal cells, cardiomyocytes, retinal epithelial cells and endothelial cells^4,5^. While the neurotoxicity of 6-OHDA results from its ability to generate quinones and reactive oxygen species and it is a widely accepted neurotoxin to model PD under in vitro and in vivo settings^6^. It is mainly taken by dopaminergic or noradrenergic neurons through their transporters and evokes cytotoxic effects by blockade of the mitochondrial respiratory chain complex I or formation of H_2_O_2_, which together lead to excessive intracellular oxidative stress^7^. Under normal conditions, free radicals or ROS are removed by various endogenous enzymatic and nonenzymatic mechanisms to promote cell survival. However, under oxidative stress conditions, a decreased expression and activities of antioxidative enzymes (superoxide dismutase, glutathione, catalase) have been reported which leads to lipid oxidization, protein inactivation, DNA damage, and cell death^8^. Thus, exogenous supplementation of antioxidants has been proposed as a potential therapy for combating the effect of increased oxidative damage.

Recently, nanoparticles with intrinsic antioxidant properties have received significant importance in nanotherapeutics to scavenge ROS in excessive inflammation and chronic injuries owing to their capability to switch between multiple oxidation states^9–11^. In particular, cerium oxide (CeO_2_) nanoparticles (CeNPs) have been reported to show neuroprotective activity because of their antioxidant and anti-apoptotic effects^12^. Lanthanides feature protective effect on neuronal cells against different forms of oxidative and nitrosative stress existing in pathological circumstances through modulation of apoptosis/survival signaling pathways and gamma‐aminobutyric acid‐activated channels^13–15^. However, major problem that still needs to be resolved for the safe and effective application of CeNPs as therapeutic agents is their agglomeration susceptibility. This is overcome by polyacrylic acid (PAA) conjugated synthesis through the chemical modification of carboxyl groups with improved chemical properties, which also facilitates targeted drug delivery^16,17^. Nanoceria can mimic the neuroprotective endogenous antioxidant enzymes since they either donate or receive electrons as they alternate between the 3+ and 4+ valence states^18^. Its usefulness in neutralizing biologically produced free radicals in the brain was evidenced by Heckman et al. in a murine model of multiple sclerosis^19^. Moreover, Elshony et al. reported the ameliorative role of cerium oxide nanoparticles against oxidative stress and apoptosis induced by insecticide fipronil in the rat brain^20^. Other in vivo study showed that cerium oxide nanoparticles ameliorated the lipid peroxidation, DNA damage and caspase-3 activity, increased antioxidant capacity and thiol molecules content and enhanced Nestin and Neurod1 mRNA expression levels in the rat brain in a model of paraquat subchronic toxicity^21^. Further, a recent article reported that ceria nanoparticles designed in a spherical shape reduced the extent of neuronal cell death and calcium dysregulation by preserving the antioxidant system in traumatic brain injury model both in vitro and in vivo^22^.

Although there are experimental studies that report the neuroprotective properties of cerium oxide^12^, there is no data on the effectiveness of PAA-conjugated cerium oxide in this respect. Herein, we investigated the neuroprotective effects of PAA-conjugated cerium oxide (PAA-CeO) nanoparticles in human neuroblastoma SH-SY5Y cells against the cell damage induced by H_2_O_2 _and 6-OHDA. Since there is still ongoing discussion about which phenotype of SH-SY5Y cells, undifferentiated or neuronal differentiated, is a more reliable neuronal-like model for neurodegeneration/neuroprotection^23^, experiments with PAA-CeO were performed in both cell phenotypes. Apart from verification of general protective effects using biochemical assays for cell viability and toxicity, we also investigated apoptotic and necrotic mechanisms as well verified the effect of synthesized nanoparticles on intracellular ROS production. Moreover, fluorescein isothiocyanate (FITC) labeled PAA-CeO was used to assess the cellular uptake of the developed nanoparticles.

## Materials and methods

### Materials

Cerium (III) nitrate hexahydrate, ammonium cerium (IV) nitrate, polyacrylic acid sodium salt (Mw = 5100), ammonium hydroxide solution (30% NH_3_ in H_2_O) and fluorescein 5-isothiocyanate were purchased from Sigma-Aldrich (Germany). For cell culture, Dulbecco's Modified Eagle Medium (DMEM), Trypsin/EDTA (0.25%) solution, heat-inactivated fetal bovine serum (FBS), Dulbecco's phosphate buffered saline (DPBS, without calcium and magnesium) and FluoroBrite™ DMEM were procured from Gibco (Invitrogen, Paisley, UK). The cytotoxicity detection kit (LDH release assay) was from Roche Diagnostic (Switzerland). Penicillin and streptomycin mixture, Triton X-100, *N*-acetyl cysteine (NAC), Ac-DEVD-CHO, propidium iodide, stabilized hydrogen peroxide solution (30% H_2_O_2_) and 6-hydroxydopamine hydrochloride were from Sigma Aldrich (Germany). Ac-DEVD-AMC, a caspase-3 fluorogenic substrate was obtained from Enzo Life Sciences (New York, NY, USA). CM-H2DCFDA assay was purchased from Molecular Probes (USA).

### Synthesis of PAA-CeO nanoparticles

The PAA-stabilized CeO_2_ nanoparticles were synthesized by low-temperature precipitation method as described previously^18^. A mixed solution containing 30 mM cerium (III) nitrate and ammonium cerium (IV) nitrate with 10% by weight of PAA were prepared and 30% ammonium hydroxide solution was added to this in dropwise manner. After continuous stirring for 24 h, the resultant solution was centrifuged at 4000 rpm for 30 min to separate out large agglomerates. The supernatant was collected and purified by dialyzing against 5 L of water at pH 7 for 2 days. The resulting solution was concentrated, sterile filtered through 20 μm sterile syringe prior to cell culture treatment. Stock solution containing PAA-CeO nanoparticles synthesized with 30 mM cerium (III) nitrate was stored in darkness at room temperature. Additionally, before the experiment it was diluted two (15 mM) and four times (7.5 mM). The final proportion of PAA-CeO nanoparticles added in the cell culture was 10% v/v stock solutions. That was denoted as 0.75 mM, 1.5 mM and 3 mM PAA-CeO.

### Characterization

The hydrodynamic particle size and zeta potential of PAA-CeO nanoparticles were determined by Dynamic Light Scattering (DLS) (Zetasizer Nano Series, Malvern Instruments). The sample was measured at 25 °C in triplicate with at least 20 measurements using water as dispersant with parameters set for cerium oxide (refractive index = 2.2 and absorption = 0.001). The particle size and morphology were visualized using Scanning Electron Microscopy (JEOL JSM-7500F, JEOL Ltd.). The X-ray Photoelectron Spectroscopy (XPS) measurements were carried out in a multi-chamber UHV system equipped with a hemispherical analyzer (SES R4000, Gammadata Scienta, Sweden) and non-monochromatized Al Kα (1486.6 eV) X-ray source. The anode was operating at 12 kV and 15 mA. Numerical analysis of the obtained spectra was carried out using CasaXPS 2.3.23 software after subtracting the Shirley-type background. The experimental results were fitted using a profile with a variable ratio (70:30) of Gaussian and Lorentzian lines^24^.

### Cell culture

Human neuroblastoma SH-SY5Y cells (ATCC CRL-2266, USA) were cultured in DMEM supplemented with a 10% v/v FBS and 1% v/v penicillin/streptomycin mixture as described previously^25^. Cells were maintained at 37 °C in a saturated, humid atmosphere containing 95% air and 5% CO_2_. After reaching 80% confluency, cells were counted manually (Bürker chamber) and seeded at a density of 4 × 10^4^, 2 × 10^5^ and 1 × 10^6^ cells per well into 96-, 24- and 6-well plates, respectively. To obtain differentiated cells (RA-SH-SY5Y), the cells were plated at a ratio of half the densities specified for undifferentiated cells and cultured in a medium supplemented with retinoic acid (RA, 10 μM) for 6 days, during which the culture medium was changed every 2 days. One day before cell treatment, the culture medium in both cell phenotypes was exchanged with DMEM containing antibiotics and 1% FBS. The cells were tested for potential Mycoplasma contamination (MycoBlue Mycoplasma Detector, VAZYME) each time the new batch of frozen cells was propagated. The cells were used for experiments between passages 5–15.

### Cell treatment

H_2_O_2_ and 6-OHDA were prepared freshly in distilled water and were added in volume 1% v/v. In similar volume (1% v/v) were added NAC (1 mM) or Ac-DEVD-CHO (20 µM), which 100 × stock solutions were prepared in DMSO and distilled water, respectively, and stored at − 20 °C. All experimental groups without nanoparticles were supplemented with vehicle (distilled water, 10% v/v). All agents were added to the culture medium at prescribed concentrations under light-limited conditions.

First, UN- and RA-SH-SY5Y cells were treated with PAA-CeO at different concentrations (0.75 mM, 1.5 mM and 3 mM) for 24 h to assess the biosafety of the nanoparticles. Next, to test the neuroprotective potential of developed nanoparticles, the cells were pretreated with PAA-CeO (0.75 mM, 1.5 mM and 3 mM) or vehicle for 30 min followed by 24 h exposure to H_2_O_2_ (at IC_50_ concentration of 0.375 and 0.5 mM for UN- and RA-SH-SY5Y, respectively) and 6-OHDA (at IC_50_ concentration of 0.1 and 0.2 mM for UN- and RA-SH-SY5Y, respectively). The optimal concentration of H_2_O_2_ was experimentally established by 24 h treatment of cells with H_2_O_2_ at concentrations 0.2–0.5 and 0.2–0.75 mM for UN- and RA-SHSY5Y cells, respectively. The optimal concentrations for 6-OHDA to induce cell damage in UN- and RA-SH-SY5Y cells were established in our previous work^26,27^. As a positive control for both oxidative stress models, an antioxidant *N*-acetyl cysteine (NAC, 1 mM) was employed, which was given concomitantly with oxidative stress inducers.

### Cell viability assay

Cell viability of UN- and RA-SH-SY5Y cells cultured and treated in 96-well plate format was estimated by biochemical WST-1 assay (Roche Diagnostic, Basel, Switzerland) as described previously^25^. The absorbance of each sample was measured 30 and 60 min after substrate addition with a multi-well plate-reader (Infinite® M200 PRO, Tecan, Switzerland) at 440 nM (measurement wavelength) and 630 nm (reference wavelength). Data (calculated difference between measurement and reference measurement) after subtraction for blank value (total damage, 1% Triton X100 for 15 min) were normalized to the vehicle-treated cells and are expressed as a percentage of the control ± SEM established from 3–4 independent experiments with 3–5 replicates each.

### Cytotoxicity assay

The level of lactate dehydrogenase (LDH) released from damaged cells into culture media, a marker of cell death, was measured using Cytotoxicity Detection Kit (Roche) as described previously^26^. Twenty four hours after cell treatment in 96-well plate format, 50 µl of the medium was transferred to a new 96-well plate where LDH reagent was added. After 15 min incubation in the dark at room temperature, the absorbance of each sample was measured with the multi-well plate-reader (Infinite® M200 PRO, Tecan, Switzerland) at 490 nm. After subtraction of blank value (absorbance of medium without cells), the data were normalized to the vehicle-treated cells (100%) and presented as a mean ± SEM established from 6-10 independent experiments with 3–5 replicates each.

### Propidium iodide staining and flow cytometry

To confirm the results obtained by the LDH release assay, we employed propidium iodide staining of the UN- and RA-SH-SY5Y cells cultured and treated in a 24-well plate format. Twenty four hours after cell treatment, the cells were collected on ice and stained with propidium iodide (PI, 10 µg/ml in DPBS) as described previously^23^. To obtain a maximal signal in PI staining, the cells were treated with TritonX_100_ for 5 min. The cells (1 × 10^4^) were analyzed in the fluorescence channel for PerCP-Cy5-5-A (red fluorescence) using BD FACS Canto II System and BD FACSDiva™ v5.0.1 Software (BD Biosciences, San Jose, CA, USA). The PI-positive cells (exhibiting loss of cell membrane integrity) represent necrotic and late apoptotic cells. Data are presented as a percentage of PI-positive cells (± SEM) established from 2-3 independent experiments with 2 replicates each.

### Microscopic assessment of morphological changes

For confirmation of biochemical results on protection mediated by PAA-CeO in UN-and RA-SH-SY5Y cells, 24 h after treatment, the cells were placed in FluoroBrite™ DMEM and were visualized by inverted fluorescence microscope (AxioObserver, Carl Zeiss). The microscopic evaluation was performed using DIC (differential interference contrast) technique and microphotographs were taken using a black–white camera (Axio-CamMRm, Carl Zeiss).

### Caspase-3 activity assay

To assess the impact of tested nanoparticles on apoptotic changes induced by H_2_O_2_ or 6-OHDA, we measured the activity of caspase-3, which is a cysteine protease and main executor of apoptosis. The UN- and RA-SH-SY5Y cells were grown in the 6-well plate and were pretreated for 30 min with PAA-CeO (3 mM) or caspase-3 inhibitor, Ac-DEVD-CHO (20 µM) followed by 9 or 18 h exposure to H_2_O_2_ and 6-OHDA, respectively. After the cell treatment, the medium was removed, and the plates with cells were stored at − 20 °C until we performed the experiment. The refrozen cells were lysed with ice-cold CAB (caspase assay buffer) supplemented with pepstatin A and leupeptin, centrifuged, and caspase-3 activity was measured in cell supernatants (placed in 96-well plates, each probe in duplicates) using fluorogenic substrate Ac-DEVD-AMC (50 µM in CAB) as described previously^27^. The data (averaged from duplicates and expressed as the mean relative fluorescence units, RFU) after blank subtraction (the signal from CAB buffer) were normalized to the protein level (measured by the BCA method) and further calculated as a percent of vehicle-treated cells and are presented as the mean ± SEM from 2-4 independent experiments with 2 replicates each.

### Estimation of ROS formation

Free radical production in UN-SH-SY5Y cells was measured with 5-(and-6)-chloromethyl-2′,7′-dichlorodihydrofluorescein diacetate acetyl ester (CM-H2DCFDA, Molecular Probes, USA) as described previously^28^. Briefly, cells cultured in 24-well plate format were loaded with 5 μM CM-H2DCFDA (in FluoroBrite™ DMEM). Next, the cells were treated with PAA-CeO (0.75–3 mM) for 15 min and exposed for the next 45 min to 0.5 mM H_2_O_2_. The antioxidant NAC (1 mM) was used as a positive control for the assay. Next, the cells were washed with pre-warmed FluoroBrite™ DMEM, collected in 1.5 ml tubes, centrifuged, and the cell pellet was resuspended in cold DPBS (without calcium and magnesium). 1 × 10^4^ cells were analyzed using BD FACS Canto II System and BD FACSDiva™ v5.0.1 Software (BD Biosciences, San Jose, CA, USA) in the fluorescence channel for FITC (green fluorescence). Mean fluorescence intensity was recorded for each sample. Data are presented as mean fluorescent intensity (± SEM) established from 2 independent experiments with 4 replicates each.

### Fluorescein isothiocyanate (FITC) labeling of PAA-CeO nanoparticles

For fluorescent labeling of PAA-CeO nanoparticles, 10 µM fluorescein isothiocyanate (FITC) solution in ethanol was suspended in 30 mM PAA-CeO and shaken in the dark at 300 rpm at room temperature overnight (Eppendorf® Thermomixer®). Particles were separated via centrifugation at 5000 rpm and washed twice with sterile double-distilled H_2_O until the supernatant turned colorless. The absorption spectra of PAA-CeO, FITC alone and FITC-PAA-CeO were measured using UV–Vis Spectrometer (Shimadzu Corporation) in the wavelength range of 200 to 800 nm.

### In vitro cellular uptake of FITC-labeled nanoparticles

To evaluate the cellular uptake of nanoparticles, cells were seeded in 24-well plates. FITC-PAA-CeO (10% v/v) at different concentrations (1.5 mM and 3 mM) were added to UN-and RA-SH-SY5Y cells for 30 min, 1 h, 2 h, 3 h and 6 h. Control cells were treated with a vehicle. After cell treatment, the cells were washed with pre-warmed FluoroBrite™ DMEM and imaged with an inverted fluorescence microscope (AxioObserver, Carl Zeiss) equipped with a black-white camera (Axio-CamMRm, Carl Zeiss). Two microphotographs (in 480 nm and DIC panels) were taken for each well. Next, the cells were collected on ice in 1.5 ml tubes, centrifuged, and the cell pellet was resuspended in cold DPBS (without calcium and magnesium). 1 × 10^4^ cells were analyzed using BD FACS Canto II System and BD FACSDiva™ v5.0.1 Software (BD Biosciences, San Jose, CA, USA) in the fluorescence channel for FITC (green fluorescence). Mean fluorescence intensity and percentage of FITC-positive cells were recorded for each sample. Data are presented as a percentage of FITC-positive cells and mean fluorescent intensity (± SEM) established from 2 independent experiments with 2 replicates each.

### Statistical analysis

The data were analyzed with Statistica 13.3 software (StatSoft Inc., Tulsa, OK, USA). One- or two-way analysis of variance (ANOVA) with post hoc Duncan’s test for multiple comparisons with assumed *P* < 0.05 were used.

## Results

### Physiochemical characteristics of synthesized PAA-CeO nanoparticles

**Figure 1:**
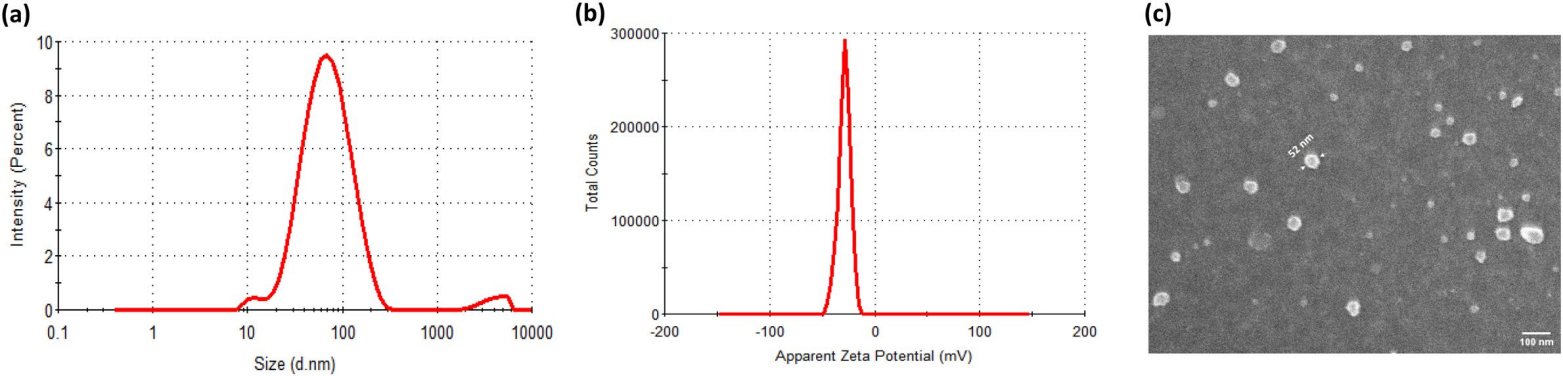
(**a**) Particle size distribution (by intensity) determined by Dynamic Light Scattering (DLS) (**b**) Zeta potential and (**c**) Scanning Electron Microscope (SEM) image of PAA-CeO nanoparticles.

PAA conjugated CeO_2_ nanoparticles were synthesized by low-temperature chemical precipitation technique. DLS data (Fig. 1a) indicated that PAA-CeO nanoparticles had narrow size distribution with particle size in the range of 50–60 nm and a polydispersity index of 0.258. Negative zeta potential of nanoparticles (− 35.0 mV) indicated their electrostatic stabilization in the aqueous phase (Fig. 1b). The stability of the nanoparticles was ensured by checking the particle size and zeta potential at regular intervals and the values displayed no statistically significant difference even after 3-months of storage (Fig. S1). The spherical morphologies of the samples were visualized using FESEM, which revealed that the average particle size is in line with DLS results (Fig. 1c).

**Figure 2:**
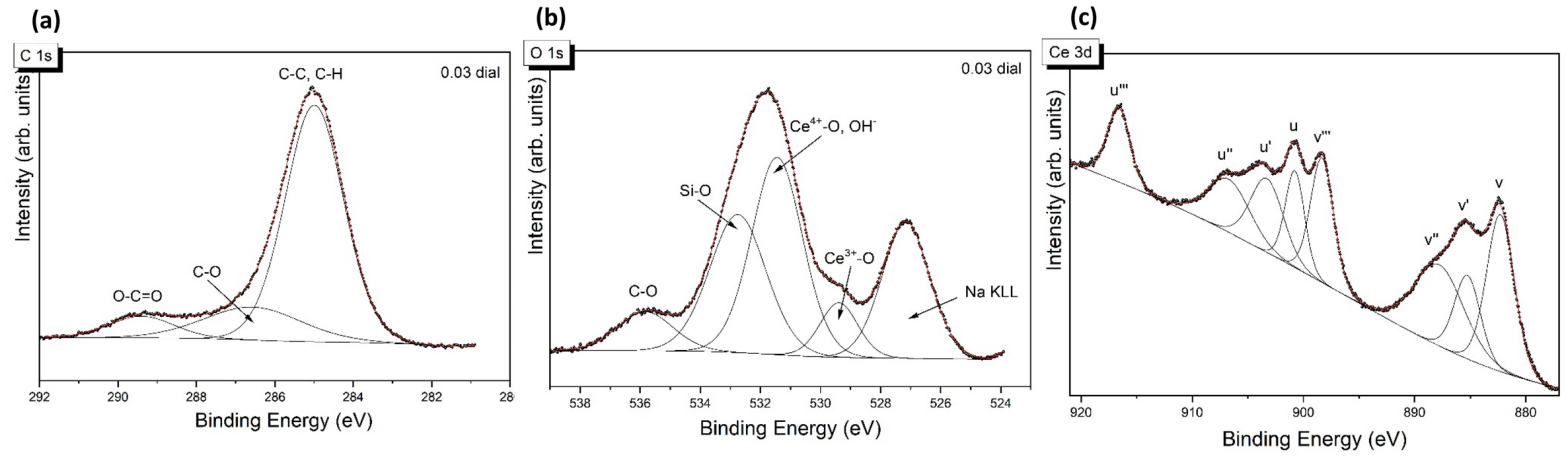
XPS spectra of (**a**) C 1 s (**b**) O 1 s and (**c**) Ce 3d lines of PAA-CeO nanoparticles.

The XPS studies revealed details on the chemical composition, chemical bonds present on the surface and the levels of oxidation of specific components. The high-resolution spectra of C 1 s, Ce 3d and O 1 s (Fig. 2) were used to identify the chemical states of the active phase in the PAA-CeO samples. Three peaks at 285.0 eV (organic contaminants), 286.0–286.6 eV (C–O groups) and 288.8–289.4 eV (O–C=O groups) can be identified in the C 1 s spectra (Fig. 2a). The O 1 s spectra (Fig. 2b) exhibit two major contributions (over 80%) located at 529.4 eV and 531.4–531.7 eV related to oxygen in Ce^4+^ and Ce^3+^ species, respectively. It is worth noting that hydroxyl groups also contribute to the component with O 1 s binding energy close to 531.5 eV. The Ce 3d spectra are presented in Fig. 2c. In accordance with the standard approach, Ce 3d spectra can be fitted with eight or ten components marked ‘v’ for Ce 3d_5/2_ and ‘u’ for Ce 3d_3/2_ multiplets. The multiplicity of these states comes from various Ce 4f. level occupancies in the final state. In this case, as it was difficult to resolve the v_0_ and u_0_ peaks due to the short energy gap between the v and u peaks, we have decided to employ an 8-components technique. The peaks at 882.5 (v), 888.1 (v″) and 898.8 eV (v′′′) as well as 901.1 (u), 906.8 (u") and 917.0 eV (u′′′) can be assigned to Ce^4+^, whereas, 885.8 (v′) and 903.9 eV (u′) to Ce^3+^. The position of Ce^4+^ reference line (u′′′) was 917.0 eV, in excellent agreement with the data in the literature^29^. The surface concentration of Ce^3+^ was determined from the Ce^3+^/(Ce^3+^  + Ce^4+^) peak areas ratio. The content of Ce^3+^ found in PAA-CeO was 21.1%. Most likely, the small amount of cerium on the surface makes it more available for exposure to oxygen and hence Ce species are more oxidized.

### Biosafety evaluation of PAA-CeO

Twenty four hours of treatment with PAA-CeO nanoparticles in the concentration range of 0.75–3 mM did not evoke any detrimental effect on UN- or RA-SH-SY5Y cells in comparison with vehicle-treated cells as was measured by LDH release assay. The LDH biochemical data were also confirmed by propidium iodide (PI) staining of cells treated for 24 h with 3 mM PAA-CeO and flow cytometry analysis, where no significant changes were observed in the number of PI-positive cells when compared to the vehicle-treated group (Table 1). Since we observed the unspecific interaction between tested nanoparticles and WST-1 assay (data not shown), we excluded this method from further studies, at least from experimental sets where PAA-CeO nanoparticles were tested.

**Table 1:** Biosafety of PAA-CeO nanoparticles in UN- and RA-SH-SY5Y cells.

Sample	UN-SH-SY5Y		RA-SH-SY5Y	
	LDH release (% Control)	PI-positive cells (%)	LDH release (% Control)	PI-positive cells (%)
Control + Vehicle	99.99 ± 0.00	13.10 ± 3.07	99.99 ± 0.00	4.96 ± 1.29
0.75 mM	99.53 ± 5.59	n.d.	97.56 ± 1.63	n.d.
1.5 mM	107.87 ± 5.63	n.d.	103.99 ± 5.39	n.d.
3 mM	107.59 ± 6.11	10.17 ± 1.04	104.52 ± 3.32	2.46 ± 0.80

The UN- and RA-SH-SY5Y cells were treated with PAA-CeO (0.75–3 mM) or vehicle for 24 h. Cell cytotoxicity was measured in a culture medium with LDH release assay and in the cells by propidium iodide (PI) staining. The LDH data were normalized to the vehicle-treated cells, whereas PI data are shown as a percentage of PI-positive cells. The data were analyzed by one-way ANOVA followed by Duncan’s posthoc test and are presented as the mean ± SEM from 4-8 independent experiments. n.d.-not determined.

### Neuroprotective effects of PAA-CeO nanoparticles against H_2_O_2_- and 6-OHDA-induced cell damage

Before testing the neuroprotective potential of synthesized nanoparticles, the reliable cell damage models were established in UN- and RA-SH-SY5Y cells using cell viability (WST-1) and toxicity (LDH release) assays (Fig. S1 and Table S1). These data confirmed our previous findings^26,27^ that concentrations of 0.375 and 0.5 mM of H_2_O_2_ for UN- and RA-H-SY5Y cells, respectively, and 0.1 and 0.2 mM 6-OHDA for UN- or RA-SH-SY5Y cells, respectively for 24 h evoke about 50% decrease in cell viability which could be prevented by co-treatment with antioxidant NAC.

**Figure 3:**
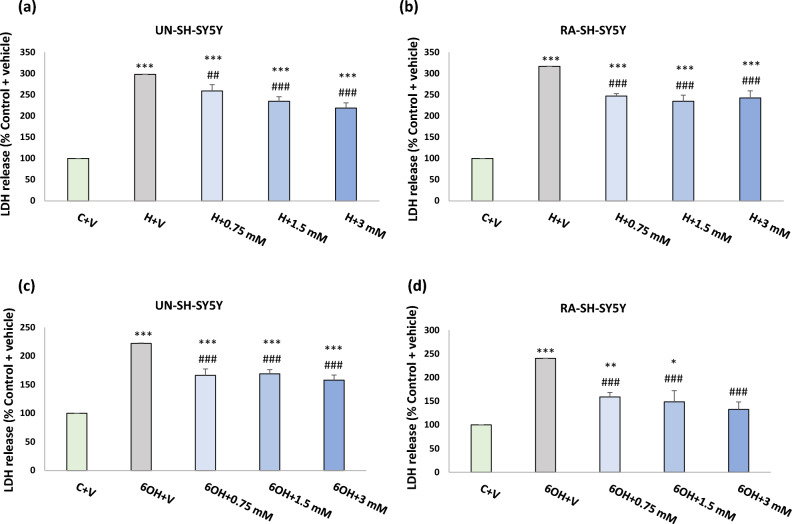
The effect of PAA-CeO on cell toxicity evoked by H_2_O_2_ (**a**, **b**) and 6-OHDA (**c**, **d**) in UN- and RA- SH-SY5Y cells. The cells were pretreated for 30 min with PAA-CeO (0.75, 1.5 and 3 mM) followed by 24 h of treatment with H_2_O_2_ (0.375 and 0.5 mM for UN- and RA-SH-SY5Y cells, respectively) or 6-OHDA (0.1 and 0.2 mM for UN- and RA-SH-SY5Y cells, respectively). The cell toxicity was measured with an LDH release assay. The data were normalized to the vehicle-treated cells, analyzed by one-way ANOVA followed by Duncan’s post hoc test and are presented as the mean ± SEM from 6-10 independent experiments. *P < 0.05, **P < 0.01 and ***P < 0.001 versus vehicle-treated cells; ^##^*P* < 0.01 and ^###^*P* < 0.001 versus H_2_O_2_/6-OHDA-treated cells. C—control, H—H_2_O_2_, 6OH—6-hydroxydopamine, N—NAC, V—vehicle.

**Figure 4:**
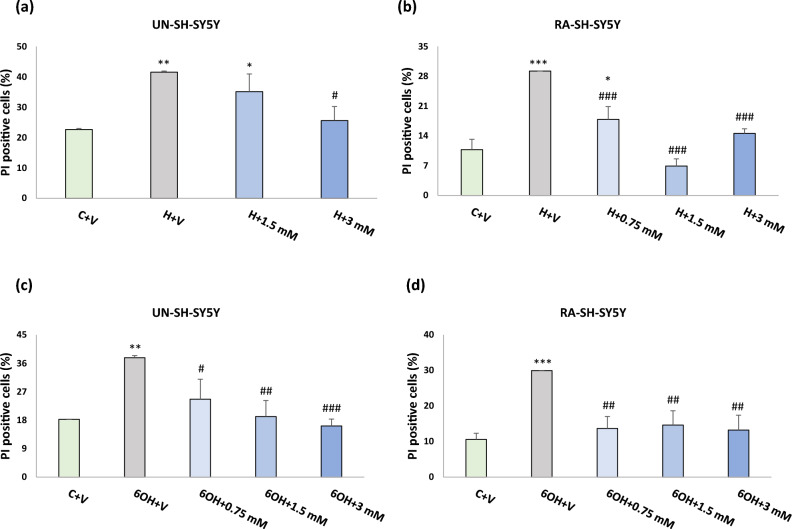
Flow cytometry analysis of propidium iodide (PI)-stained cells in UN- and RA-SH-SY5Y cells. The cells were pretreated for 30 min with PAA-CeO (0.75, 1.5 and 3 mM) followed by 24 h of treatment with H_2_O_2_ (**a**, **b**; 0.375 and 0.5 mM for UN- and RA-SH-SY5Y cells, respectively) or 6-OHDA (**c**, **d**; 0.1 and 0.2 mM for UN- and RA-SH-SY5Y cells, respectively). The cells were stained with propidium iodide (PI) and measured by flow cytometry. Data from 2–3 independent experiments with 2 repetitions each were analyzed by one-way ANOVA followed by Duncan’s post hoc test and are presented as the mean ± SEM of PI-positive cells. ^*^P < 0.05, **P < 0.01 and ***P < 0.001 versus vehicle-treated cells; ^#^*P* < 0.05, ^##^*P* < 0.01 and ^###^*P* < 0.001 versus H_2_O_2_/6-OHDA-treated cells. C—control, H—H_2_O_2_, 6OH—6-hydroxydopamine, N—NAC, V—vehicle.

**Figure 5:**
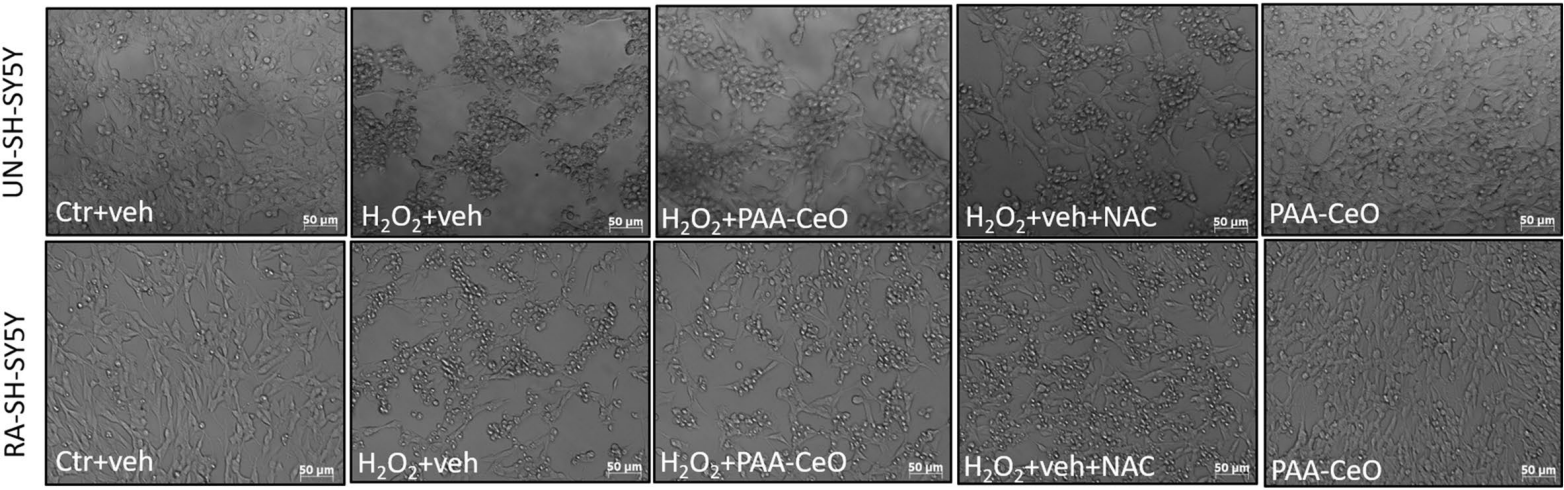
Representative DIC (*Differential interference contrast*) microphotographs of UN- and RA- SH-SY5Y cells pretreated for 30 min with PAA-CeO (3 mM) followed by 24 h of incubation with H_2_O_2_ (0.375 and 0.5 mM for UN-and RA-SH-SY5Y cells, respectively). An antioxidant *N*-acetyl-cysteine (NAC, 1 mM) was used as the positive control to this oxidative stress model.

**Figure 6:**
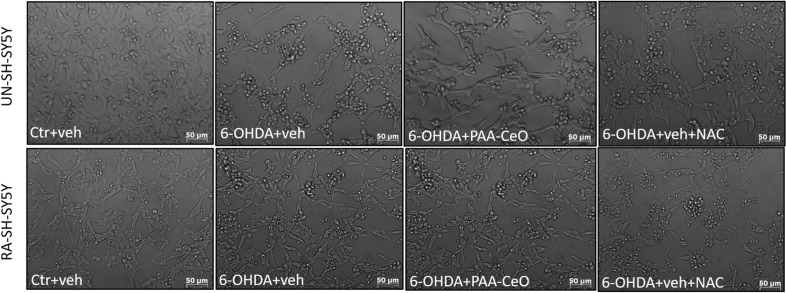
Representative DIC (*Differential interference contrast*) microphotographs of UN- and RA- SH-SY5Y cells pretreated for 30 min with PAA-CeO (3 mM) followed by 24 h incubation with 6-OHDA (0.1 and 0.2 mM for UN-and RA-SH-SY5Y cells, respectively). An antioxidant *N*-acetyl-cysteine (NAC, 1 mM) was used as the positive control to this oxidative stress model.

In order to test the neuroprotective potential of PAA-CeO, the UN- and RA-SH-SY5Y cells were pretreated with different concentrations of nanoparticles (0.75, 1.5 and 3 mM) for 30 min followed by 24 h exposure to cell damaging factors (H_2_O_2_ or 6-OHDA). We observed an almost threefold increase in cytotoxicity after incubation of UN-SH-SY5Y cells with H_2_O_2_ (0.375 mM) when compared to vehicle-treated cells, which was substantially reduced by all tested concentrations of PAA-CeO (0.75, 1.5 and 3 mM) (Fig. 3a). Similarly, in RA-SH-SY5Y cells H_2_O_2_ (0.5 mM) evoked over threefold increase in LDH release which was significantly attenuated by all tested concentration of PAA-CeO (Fig. 3b). In the model of 6-OHDA-induced cell damage in UN-SH-SY5Y we observed over twofold increase in LDH release after exposure to 0.1 mM 6-OHDA which was substantially reduced by all tested concentration of PAA-CeO (Fig. 3c). Similarly, an almost 2.5-fold increase of cytotoxicity was observed in RA-SH-SY5Y cells incubated for 24 h with 0.2 mM 6-OHDA, which was also significantly reduced by all tested concentrations of PAA-CeO (Fig. 3d). Further, the neuroprotective potential of PAA-CeO was confirmed by PI staining in both cell phenotypes. We found that PAA-CeO at various concentrations partially attenuated the number of damaged (PI-positive) nuclei induced by H_2_O_2_ (Fig. 4a,b) or 6-OHDA (Fig. 4c,d). Moreover, protection mediated by PAA-CeO nanoparticles in both cell damage models in UN- and RA-SH-SY5Y cells were also evidenced by light microscopy (DIC imaging) (Figs. 5, 6).

### The effect of PAA-CeO on caspase-3 activity in UN- and RA-SH-SY5Y cells

Based on our previous studies which showed the participation of apoptosis measured by caspase-3 activation in the mechanism of H_2_O_2_ or 6-OHDA-evoked cell damage in UN- and RA-SH-SY5Y cells^27^, we tested the effect of PAA-CeO on this apoptotic cell death marker. We observed activation of caspase-3 in both models of cell damage (after 9 and 18 h of treatment with H_2_O_2_ and 6-OHDA, respectively) in UN- and RA-SH-SY5Y cells which was totally prevented by caspase-3 inhibitor Ac-DEVD-CHO (Tables 2 and 3). That is in line with our previous reports^25,27^. Although PAA-CeO (3 mM) alone significantly inhibited the basal caspase-3 activity after 9 and 18 h of incubation with RA- (Table 2) and UN- (Table 3) SH-SY5Y cells, respectively, it did not affect the H_2_O_2_-induced caspase-3 activity in both cell phenotypes. However, we observed partial attenuation by PAA-CeO (3 mM) of the 6-OHDA-induced this apoptotic protease activity in UN- and RA-SH-SY5Y cells (Table 3).

**Table 2:** Effects of PAA-CeO on the H_2_O_2_-induced caspase-3 activity in UN- and RA-SH-SY5Y cells.

Sample	UN-SH-SY5Y 9 h	RA-SH-SY5Y 9 h	RA-SH-SY5Y 18 h
Control + Vehicle	100.00 ± 0.00	100.00 ± 0.00	100 ± 0.00
H_2_O_2_ + Vehicle	344.46 ± 0.00***	185.48 ± 0.00***	699.60 ± 2.40***
H_2_O_2_ + PAA-CeO	342.52 ± 25.11***	182.07 ± 17.85***	734.42 ± 63.17***
H_2_O_2_ + Ac-DEVD-CHO	2.94 ± 0.87***, ###	1.92 ± 0.77***, ###	n.d.
PAA-CeO	77.56 ± 12.08	33.06 ± 4.77***	n.d.

The UN- and RA-SH-SY5Y cells were pretreated for 30 min with PAA-CeO (3 mM) followed by 9 h of treatment with H_2_O_2_ (0.375 and 0.5 mM for UN- and RA-SH-SY5Y, respectively). For RA-SH-SY5Y cells caspase-3 activity was also measured 18 h after H_2_O_2_ (0.5 mM) treatment. Ac-DEVD-CHO (20 μM) was used as a positive control for the assay. Data were normalized to vehicle-treated cells (control) and are presented as the mean ± SEM from 2–3 independent experiments with 2 replicates each. ***P < 0.001 versus vehicle-treated cells; ^###^*P* < 0.001 versus H_2_O_2_-treated cells. n.d.-not determined.

**Table 3:** Effects of PAA-CeO on the 6-OHDA-induced caspase-3 activity in UN- and RA-SH-SY5Y cells.

Sample	UN-SH-SY5Y	RA-SH-SY5Y
Control + Vehicle	100.00 ± 0.00	100.00 ± 1.83
6-OHDA + Vehicle	238.67 ± 0.21***	405.49 ± 1.17***
6-OHDA + PAA-CeO	217.36 ± 4.71***, #	299.26 ± 41.63***, ##
6-OHDA + Ac-DEVD-CHO	2.50 ± 0.86***, ###	6.67 ± 0.68**, ###
PAA-CeO	52.83 ± 13.50***	83.74 ± 14.14

The UN- and RA-SH-SY5Y cells were pretreated for 30 min with PAA-CeO (3.0 mM) followed by 18 h of treatment with 6-OHDA (0.1 and 0.2 mM for UN- and RA-SH-SY5Y, respectively). Ac-DEVD-CHO (20 μM) was used as a positive control for the assay. Data were normalized to vehicle-treated cells (control) and are presented as the mean ± SEM from 2-4 independent  experiments with 2 replicates. **P < 0.01 and ***P < 0.001 versuss vehicle-treated cells; ^#^*P* < 0.05, ^##^*P* < 0.01 and ^###^*P* < 0.001 versus 6-OHDA-treated cells.

### Effects of PAA-CeO on the H_2_O_2_-induced ROS formation in UN-SH-SY5Y cells

**Figure 7:**
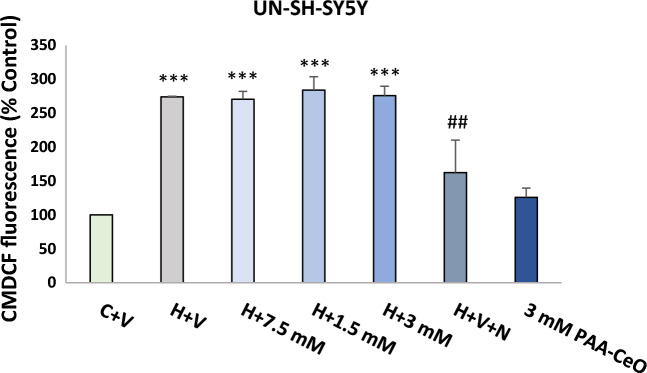
Effect of PAA-CeO on the H_2_O_2_-evoked increase in reactive oxygen species (ROS) level in UN-SH-SY5Y cells. The cells were loaded with 5 μM of CM-H2DFFDA, incubated with PAA-CeO (0.75–3 mM) for 15 min, followed by 45 min exposure to 0.5 mM H_2_O_2_. An antioxidant *N*-acetylcysteine (NAC, 1 mM) was used as a positive control for the assay. The cells were analyzed by flow cytometry and data are presented as a mean CM-DCF fluorescence ± SEM from 2 independent experiments with 4 replicates each. ***P < 0.001 versus vehicle-treated cells; ^##^*P* < 0.01 versus H_2_O_2_-treated cells. C—control, H—H_2_O_2_, N—NAC, V—vehicle.

Based on our previous data, which demonstrated increased intracellular ROS in the cell damaging effect of H_2_O_2_ in SH-SY5Y cells^28^ we tested the effect of PAA-CeO on this parameter using fluorescent intracellular ROS probe CM-H2DCFDA. Similar to our previous study, we found about a threefold increase in CM-DCF fluorescence after H_2_O_2_ (0.5 mM for 45 min) when compared with the vehicle-treated cells^28^, which was entirely reduced by antioxidant NAC (1 mM) but not changed by PAA-CeO at all tested concentrations (Fig. 7).

### Nanoparticle labeling with FITC

**Figure 8:**
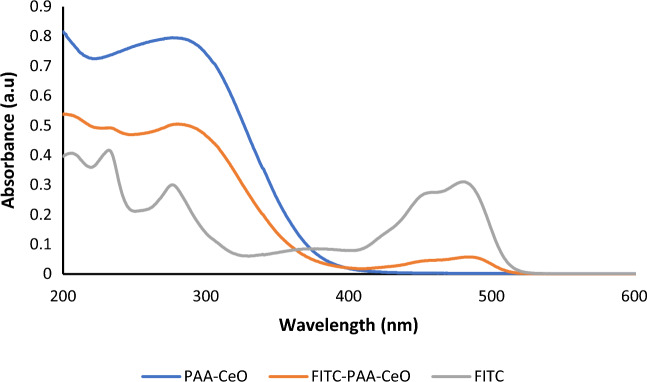
UV–Vis absorption spectra of pure FITC (grey), PAA-CeO (blue) and FITC labeled PAA-CeO nanoparticles (orange).

Flow cytometry enables the quantification of cell uptake of nanoparticles with their light scattering and fluorescence signals. Since pure PAA-CeO NPs are not fluorescent in nature, it was necessary to conjugate a fluorescent label to impart luminescence. Therefore, a standard fluorescence labeling molecule, FITC, was conjugated with the nanoparticle. Figure 8 shows the absorption spectra of PAA-CeO alone and FITC labeled PAA-CeO compared to pure FITC. Fluorescein isothiocyanate absorbs most efficiently at its peak or excitation maximum of 490 nm, similar to previous literature reports^30^. The absorbance peak was observed at the wavelength of 300 nm, corresponding to the characteristic absorption peak of Ce^4+^ of CeO_2_ nanoparticles^31^. While FITC labeled PAA-CeO exhibited bands corresponding to FITC around 490 nm as well as for Ce^4+^ around 302 nm. Thus, the absorption spectrum depicts the successful loading of FITC in PAA-CeO.

### Cell uptake of FITC labeled PAA-CeO nanoparticles

**Figure 9:**
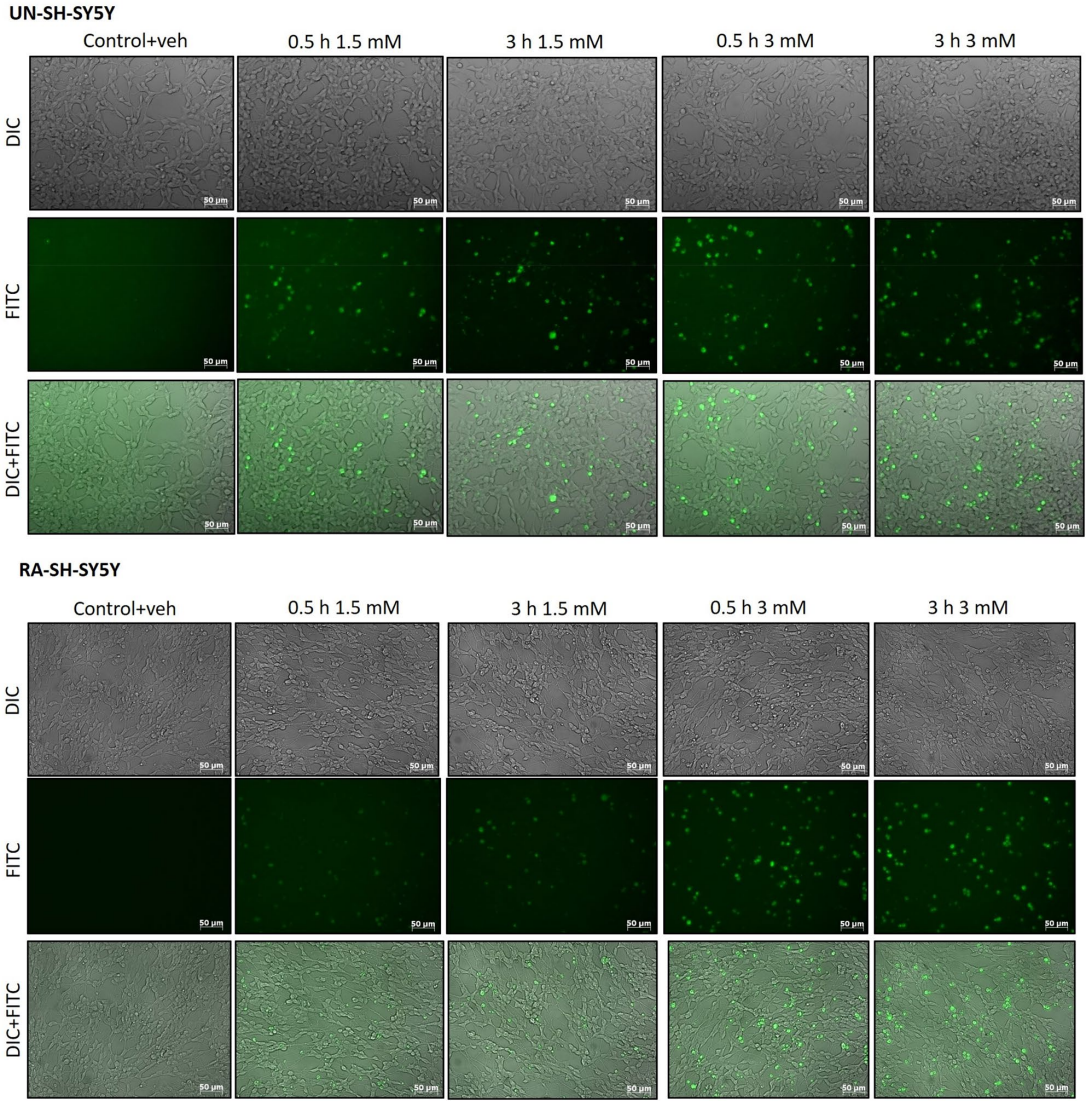
Representative images (green and DIC channels) of cellular uptake of FITC-labelled PAA-CeO nanoparticles (1.5 and 3 mM) after incubation for 30 min and 3 h in (**a**) UN- and (**b**) RA- SH-SY5Y cells.

**Figure 10:**
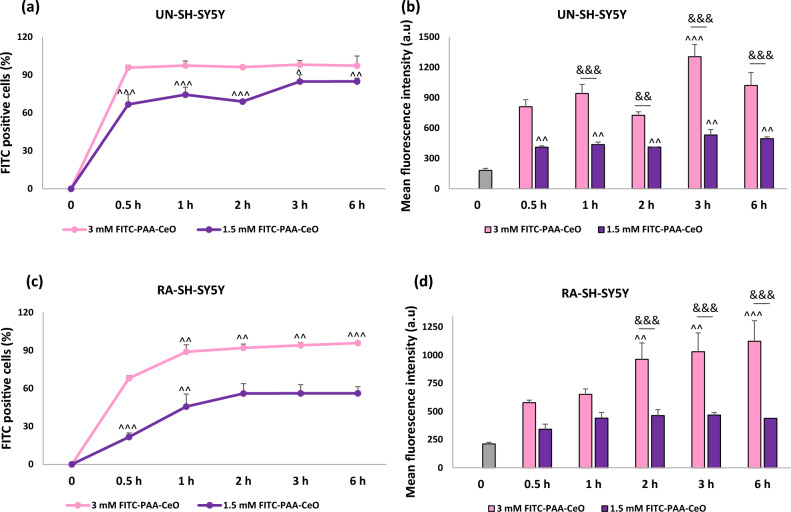
The percentage of FITC-positive cells (**a**, **b**) and Mean fluorescence intensity of FITC-labelled PAA-CeO (**c**, **d**) nanoparticles after incubation in UN- (**a**, **c**) and RA- (**b**, **d**) SH-SY5Y cells for 30 min, 1 h, 2 h, 3 h and 6 h, respectively. Control cells were treated with a vehicle (time point 0). Data are presented as the mean ± SEM from 2 separate experiments with 2 replicates each. ^*P* < 0.05, ^^*P* < 0.01 and ^^^*P* < 0.001 versus 3 mM at 30 min; ^&&^*P* < 0.01 and ^&&&^*P* < 0.001 1.5 versus 3 mM FITC-PAA-CeO for particular time point.

Nanoparticle cellular uptake refers to their ability to penetrate the cell membrane for which we used FITC-labeled PAA-CeO at different concentrations (1.5 and 3 mM) that were added to cells for up to 6 h. In vitro cellular uptake of FITC-PAA-CeO nanoparticles was performed using an inverted fluorescence microscope for visual representation and qualitative analysis. We observed a time- and concentration-dependent increase in fluorescence signal in both cell phenotypes (Fig. 9). The flow cytometry analysis of FITC-labeled PAA-CeO demonstrated that the number of FITC positive cells was increased as a function of time and the signals were dependent on their concentration as well. After 6 h of cell incubation with stained nanoparticles, approx. 97% and 84% of UN-SH-SY5Y cells were FITC-positive at concentrations of 1.5 and 3 mM, respectively, which was not distinctly different from the result after 6 h of incubation in RA- SH-SY5Y cells (Fig. 10a,b). Additionally, the uptake of FITC-PAA-CeO was analyzed at the level of median fluorescence intensity of the FITC signal where 3 mM FITC-PAA-CeO unveiled maximum mean fluorescence intensity after 3 h of incubation in UN-SH-SY5Y cells. In contrast, in RA-SH-SY5Y cells, 3 mM exhibited an increasing trend following the treatment and the maximum value was attained at 6 h (Fig. 10c,d). These data point to similar internalization of FITC-PAA-CeO into both cell phenotypes, which depends on the concentration of tested nanoparticles.

## Discussion

Most neurodegenerative diseases are associated with increased oxidative stress in the brain^1^. Based on the prominent behavior of cerium oxide as a regenerative antioxidant^12^, it was justified to study the neuroprotective effects of PAA-CeO nanoparticles against cell damage induced by H_2_O_2_ and 6-OHDA which, as reported previously, mechanistically induce elevated oxidative stress which is detrimental to neuronal cells^25–28^. Due to their poor water solubility, low targeting effectiveness and toxicity concerns, the applicability of inorganic nanoparticles such as cerium oxide in its pure form is often considered to be limited^32^. Without the conjugation of additional molecular moiety, these nanoparticles usually exhibit a nonselective distribution across the body and exhibit poor colloidal stability. Therefore, their surface properties need to be modified by conjugating specific surface groups, polymers, or biomolecules to overcome these shortcomings^33,34^. In this study, we have successfully synthesized PAA-CeO nanoparticles in the 50–60 nm range with a zeta potential in water of − 35 mV. The effectiveness of the nanoparticle system is also associated with its stability^35^. We observed that the developed PAA-CeO nanoparticle possessed excellent stability without aggregation for up to 3 months (Fig. S1), making them suitable for biomedical applications. In the PAA-CeO nanoparticles, cerium oxide exists in mixed oxidation states of Ce^3+^ and Ce^4+^, as evidenced by XPS spectra (Fig. 2). The coexistence of two oxidation states enables redox reactions where Ce^3+^ is oxidized to Ce^4+^ and H_2_O_2_ is formed when nanoceria reduces superoxide radicals^36^. Ce^4+^ can also oxidize H_2_O_2_ to O_2_ and regenerate Ce^3+^^37^. That is a useful way of regenerating reduced nanoceria and removing ROS, which could be beneficial for neuroprotective effects, especially in the models engaging oxidative stress inducers^38–40^. Liu et al. have proved that ROS induced in J774A.1 macrophage cells by exposure to H_2_O_2_ (0.5 mM for 15–20 min) were significantly scavenged by the CeO_2_ at concentrations of 0.5 and 1 μg/mL^18^. In an effort to lessen the aberrant generation of ROS resulting from mitochondrial dysfunction, Kwon et al. synthesized triphenylphosphonium-conjugated ceria nanoparticles (TPP-ceria NPs) that were safe for cells and attenuated mitochondrial ROS^41^. However, in our study, we excluded the direct effect of PAA-CeO on intracellular ROS production (Fig. 7), suggesting an involvement of other intracellular mechanisms in the neuroprotective effects of these polymer-conjugated nanoparticles.

The capability of nanoparticles to penetrate the cell membrane is often referred to as cellular uptake. It was necessary to do an additional labeling step since unmodified PAA-CeO are not fluorescent. Hence, a standard fluorescence labeling molecule, FITC, was consequently adsorbed onto the surface of the nanoparticles. A similar conjugation technique has been applied earlier by Jochums et al. to investigate the uptake of titanium oxide nanoparticles in human lung carcinoma (A549) and mouse fibroblast (NIH/3T3) cells^42^. Our results showed the relatively fast cellular uptake of FITC-PAA-CeO in SH-SY5Y cells, which was rather not affected by the differentiation state of the cells. These data extend the usefulness of SH-SY5Y cells as a neuronal-like model for studies on neuroprotective potency not only for polyelectrolyte-coated nanocapsules^43^ but also for polymer-conjugated cerium oxide nanoparticles.

Most research reports have demonstrated the evident utility of cerium oxide systems in neurodegenerative diseases at therapeutic doses, in which cerium oxide did not exhibit any toxicity at a wide range of concentration^18–22^. We showed for the first time that PAA-CeO is neuroprotective against H_2_O_2_- and 6-OHDA-induced cell damage in neuronal cells, and this effect was associated with the inhibition of necrosis in both tested models of cell damage (H_2_O_2_ and 6-OHDA). Since in our experimental model, during the cell treatment, we used a cell culture medium with 1% FBS, which could slow down the cell proliferation and sensitize cells (serum deprivation) to harmful stimuli, it is not excluded that necrotic processes predominated. Previous experimental data on cerium oxide-mediated brain protection implicated the inhibition of apoptosis among mechanisms of its beneficial effects^20,21^. Since we used models of neuronal cell damage models that engage mixed apoptotic-necrotic mechanisms^26,27^, we attempted to verify how PAA-CeO affects the H_2_O_2_- and 6-OHDA -stimulated caspase-3 activity. In both models of cell damage (H_2_O_2_ and 6-OHDA), we showed participation of necrosis and apoptosis when their contribution to cell death probably is not equal. Thus in H_2_O_2_ model, when probably necrosis predominates, we did not find any effect of PAA-CeO on caspase-3 activity, whereas in 6-OHDA model we showed partial reduction of this enzyme activity by tested nanoparticles. These data rather suggest a marginal role of the inhibition of caspase-3 as a main mechanistic factor in the PAA-CeO-mediated neuroprotection, at least in our human neuronal-like model. However, at the basal level of caspase-3 activity, we observed the inhibitory effect of PAA-CeO alone, proving that this compound could attenuate apoptotic changes. Although several studies revealed the protective efficacy of cerium oxide systems against oxidative damage in vitro, the mechanism of protection between apoptotic and necrotic cell death is still unknown. Most reported studies are focused on neuroprotection against apoptotic cell damage, and no previous studies demonstrated the apoptotic-necrotic mechanisms in cerium oxide systems. Intracellular protection mechanisms against oxidative free radical damage include scavenging enzyme systems, which lower the level of ROS, such as superoxide dismutase (SOD), catalase and glutathione peroxidase (GPx)^44^. Therefore, our future work will rely on these enzymes to better understand how these scavenging enzymes control H_2_O_2_ and 6-OHDA-induced cell damage. There is also further potential to study the involvement of other signaling pathways like the activity of calcium-dependent cysteine protease, calpain; caspase-3 independent apoptosis, lysosomal enzymes (e.g., cathepsin D), or intracellular pro-survival pathways like PI3-K/Akt and ERK1/2^25–27^.

## Conclusion

The stable PAA-CeO nanoparticles have been successfully developed and showed relatively fast cellular uptake in UN- and RA- SH-SY5Y cells. The cytotoxicity assay of the developed particles confirms their biosafety to both phenotypes of human neuroblastoma SH-SY5Y cells. The 30 min pre-treatment of UN- and RA-SH-SY5Y cells with PAA-CeO reduced the extent of cell damage evoked by H_2_O_2_ and 6-OHDA, demonstrating pronounced neuroprotective potential. Moreover, the mechanisms of this protection in both models are rather associated with the inhibition of necrotic processes and the model-dependent attenuation of activity of executor apoptotic protease, caspase-3 (6-OHDA model) but not with the direct inhibition of ROS (H_2_O_2_ model). The obtained data supports further investigation on the mechanisms of PAA-CeO neuroprotection and their efficacy in in vivo models and could be an ideal candidate for further development for theranostic application. The additional advantage of PAA-CeO nanoparticles is the possibility of their further functionalization, e.g., with PEG chains to avoid fast clearance or with targeting ligands.

### Supplementary Information

Supplementary Information.

## Data Availability

The datasets used and analyzed during the current study are available from the corresponding author on reasonable request.
