# Supplementary material for: Neuroprotective effects of polyacrylic acid (PAA) conjugated cerium oxide against hydrogen peroxide- and 6-OHDA-induced SH-SY5Y cell damage
Source: Sci Rep. 2023 Oct 28;13:18534. doi: 10.1038/s41598-023-45318-6 (PMC10613241; doi:10.1038/s41598-023-45318-6)
Supplement: Supplementary file 1 — Supplementary Information. [file 41598_2023_45318_MOESM1_ESM.docx]

**Supplementary Information**

**Neuroprotective effects of polyacrylic acid (PAA) conjugated cerium oxide against hydrogen peroxide- and 6-OHDA-induced SH-SY5Y cell damage**

Rugmani Meenambal^1^,Tomasz Kruk^2^, Jacek Gurgul^2^, Piotr Warszyński^2^ and Danuta Jantas^1*^

*^1^ Department of Experimental Neuroendocrinology, Maj Institute of Pharmacology, Polish Academy of Sciences, Krakow, Poland*

*^2^Jerzy Haber Institute of Catalysis and Surface Chemistry, Polish Academy of Sciences, Krakow, Poland*

*corresponding author: [jantas@if-pan.krakow.pl](mailto:jantas@if-pan.krakow.pl) (D.Jantas)

**Stability studies**

The effectiveness of the nanoparticles can only be ensured when the formulation is stable enough. The long-term study was performed to evaluate the stability of PAA-CeO, which should result in minimum aggregation over an extended period of storage at room temperature in limited light conditions. In some cases, upon storage, nanoparticles tend to aggregate which results in uneven size distribution and overall poor stability of the nanoparticles. Here, the size distribution and zeta potential at regular intervals of 15 days up to 90 days displayed no statistically significant difference. This study indicated that the developed PAA-CeO nanoparticles are highly stable at room temperature.

**Fig. S1.** The size distribution and zeta potential of PAA-CeO nanoparticles stored for three-months at room temperature

**Toxicity curves for H_2_O_2_ in UN- and RA-SH-SY5Y**

Twenty-four hours of treatment UN-SH-SY5Y with H_2_O_2_ at concentrations 0.2-0.5 mM evoked a concentration-dependent decrease in cell viability (WST-1 assay), which was prevented by concomitant treatment with an antioxidant N-acetyl-cysteine (NAC, 1 mM) (**Fig. S2a**). This reduction in cell viability evoked by H_2_O_2_ was associated with the concentration-dependent increase of LDH level in the culture medium (**Fig. S2b**). In RA-SH-SY5Y cells, the treatment with H_2_O_2_ (0.25-0.75 mM) for 24 h also induced a concentration-dependent reduction in cell viability which was reduced by NAC (**Fig S2c**) as well as evoked an increase in LDH release (**Fig. S2d**). On the basis of the cell viability data, for further neuroprotection studies, the chosen concentrations were H_2_O_2_ at 0.375 and 0.5 mM for UN- and RA-SH-SY5Y cells, respectively.

**Figure S2.** Toxicity curves for H_2_O_2_ in UN- and RA-SH-SY5Y cells. The cells were treated for 24 h with H_2_O_2_ (0.2-0.5 and 0.25-0.75 mM for UN- and RA-SH-SY5Y cells, respectively) alone or in combination with antioxidant N-acetyl-cysteine (NAC, 1 mM) followed by cell viability (WST-1 assay) and cytotoxicity (LDH release assay) measurements. The data (normalized to vehicle-treated cells) from 2-6 independent experiments are presented as the mean ± SEM. They were analyzed by two- (WST-1 assay) or one-way (LDH assay) ANOVA followed by Duncan’s posthoc test. ^*^P<0.05, ^**^P<0.01 and ^***^P<0.001 *vs.* vehicle treated cells; ^##^P<0.01 and ^###^P<0.001 vs. H_2_O_2_-treated cells.

**Effect of antioxidant NAC on cell damage induced by 6-OHDA**

Twenty-four hours of treatment in UN- and RA-SH-SY5Y with 6-OHDA at concentrations of 0.1 and 0.2 mM, respectively, evoked significant cell damage, which was attenuated by concomitant treatment with antioxidant NAC (1 mM). **Table S1** shows the effect of NAC on cell damage induced by 6-OHDA in both cell phenotypes as measured by cell viability (WST-1) and cytotoxicity (LDH release) assays.

**Table S1.** Effects of antioxidant NAC against the 6-OHDA-evoked cell damage in UN- and RA-SH-SY5Y cells

| **Sample** | **UN-SH-SY5Y** | | **RA-SH-SY5Y** |
| --- | --- | --- | --- |
|  | **LDH release**  **(% Control)** | **WST-1**  **(% Control)** | **LDH release**  **(% Control)** |
| Control+Vehicle | 99.99 ± 0.00 | 99.99 ± 0.00 | 99.64 ± 0.35 |
| 6-OHDA+Vehicle | 281.87 ± 7.78 ^***^ | 62.67 ± 3.91 ^**^ | 271.79 ± 3.03 ^***^ |
| 6-OHDA+NAC+Vehicle | 212.57 ± 15.95 ^***, ##^ | 94.44 ± 7.08 ^##^ | 188.70 ± 21.75 ^***, ##^ |

The cells were treated for 24 h with 6-OHDA (0.1 and 0.2 mM for UN- and RA-SH-SY5Y cells, respectively) alone or in combination with antioxidant N-acetyl-cysteine (NAC, 1 mM) followed by cell viability (WST-1 assay) and cytotoxicity (LDH release assay) measurements. The data from 3-4 independent experiments were normalized to vehicle-treated cells and analyzed by one-way ANOVA followed by Duncan’s post hoc test. ^**^P<0.01 and ^***^P<0.001 vs. vehicle treated cells; ^##^P<0.01 vs. 6-OHDA-treated cells.
